# Systemic effects in naïve mice injected with immunomodulatory lectin ArtinM

**DOI:** 10.1371/journal.pone.0187151

**Published:** 2017-10-30

**Authors:** Patrícia Kellen Martins Oliveira Brito, Thiago Eleutério Gonçalves, Fabrício Freitas Fernandes, Camila Botelho Miguel, Wellington Francisco Rodrigues, Javier Emílio Lazo Chica, Maria Cristina Roque-Barreira, Thiago Aparecido da Silva

**Affiliations:** 1 Departamento de Biologia Celular e Molecular e Bioagentes Patogênicos, Faculdade de Medicina de Ribeirão Preto, Universidade de São Paulo, Ribeirão Preto, SP, Brazil; 2 Curso de Pós-Graduação em Ciências da Saúde da Universidade Federal do Triângulo Mineiro, Uberaba, MG, Brazil; National Institute of technology Rourkela, INDIA

## Abstract

Toll-like receptors (TLR) contain N-glycans, which are important glycotargets for plant lectins, to induce immunomodulation. The lectin ArtinM obtained from *Artocarpus heterophyllus* interacts with TLR2 N-glycans to stimulate IL-12 production by antigen-presenting cells and to drive the immune response toward the Th1 axis, conferring resistance against intracellular pathogens. This immunomodulatory effect was demonstrated by subcutaneously injecting (s.c.) ArtinM (0.5 μg) in infected mice. In this study, we evaluated the systemic implications of ArtinM administration in *naïve* BALB/c mice. The mice were s.c. injected twice (7 days interval) with ArtinM (0.5, 1.0, 2.5, or 5.0 μg), LPS (positive control), or PBS (negative control) and euthanized after three days. None of the ArtinM-injected mice exhibited change in body weight, whereas the relative mass of the heart and lungs diminished in mice injected with the highest ArtinM dose (5.0 μg). Few and discrete inflammatory foci were detected in the heart, lung, and liver of mice receiving ArtinM at doses ≥2.5 μg. Moreover, the highest dose of ArtinM was associated with increased serum levels of creatine kinase MB isoenzyme (CK-MB) and globulins as well as an augmented presence of neutrophils in the heart and lung. IL-12, IFN-γ, TNF-α, and IL-10 measurements in the liver, kidney, spleen, heart, and lung homogenates revealed decreased IL-10 level in the heart and lung of mice injected with 5.0 μg ArtinM. We also found an augmented frequency of T helper and B cells in the spleen of all ArtinM-injected *naïve* mice, whereas the relative expressions of T-bet, GATA-3, and ROR-γt were similar to those in PBS-injected animals. Our study demonstrates that s.c. injection of high doses of ArtinM in *naïve* mice promotes mild inflammatory lesions and that a low immunomodulatory dose is innocuous to *naïve* mice.

## Introduction

Lectins are characterized as a group of proteins that interact with specific carbohydrates in a reversible and non-catalytic manner [1]. The recognition of mono- or oligosaccharides by lectins, which are ubiquitous in nature, accounts for several biological activities [2–6]. Recently, plant lectins have being explored owing to their ability to modulate immune responses in mammals. The modulation process is initiated by the interaction of lectin with glycans that are linked to receptors on the surface of adaptive and innate immunity cells [7–10]. Toll-like receptors (TLR) are importantly implicated in the modulation of innate immune response against several pathogens [11]; N-glycans exhibited by TLR on antigen-presenting cells (APC) can be recognized by plant lectins to trigger cell activation [12]. The lectin ArtinM, obtained from the seeds of *Artocarpus heterophyllus*, interacts with glycans N-linked to TLR2 [7], CD3 [13, 14], and CXCR2 [15], and these established interactions are biologically relevant since they induce activation of APC, CD4^+^ T cells (also known as T helper cells), and neutrophils, respectively [13–17]. ArtinM is organized as a homotetramer composed of 16 kDa non-glycosylated subunits [17], and each polypeptide chain encompasses a carbohydrate recognition domain (CRD). It recognizes Manα1–3 [Manα1–6] Manβ1–4, the core of N-glycans, as demonstrated by assaying the affinity of lectin to bind to a broad array of glycans [18, 19].

ArtinM induces neutrophil migration, superoxide production, and phagocytic activity [15–17]; mast cell degranulation and release of mediators such as TNF-α [20]; activation of spleen cells and CD4^+^ T cells [14]; stimulation of the interleukin (IL)-17 production by CD4^+^ T cells [13]; and IL-12 production by macrophages and dendritic cells [7, 21]. ArtinM-induced IL-12 production drives the immune response toward the T helper (Th) 1 cells [7, 21, 22], a phenomenon demonstrated in murine models of infections with *Leishmania major* [8], *L*. *amazonensis* [10], *Paracoccidioides brasiliensis* [21, 22], *Candida albicans* [23], and *Neospora caninum* [24].

A crescent number of TLR agonists are being reported as being able to elicit innate immune responses toward an inflammatory pattern, acting as immunomodulatory agents, and providing interesting tools of interference in the outcome of infections, particularly those caused by intracellular pathogens [9, 11, 25–27]. TLR agonists may exert inflammatory activities at the site of infection and aggravate tissue damage [28, 29]. Therefore, to avoid exacerbated inflammation, responses triggered by TLR agonists must be tightly regulated. Concerning TLR2, some studies support the idea that this receptor plays both proinflammatory and regulatory roles [30, 31]. However, pre-clinical assays for the applications of TLR agonists as immunomodulatory agents require *in vivo* approaches, including quantitative analysis of tissue damage after agonist administration. Furthermore, all the possible collateral effects of a TLR agonist administration must be examined in a large spectrum of conditions to which the treated organism is exposed.

The TLR2 agonist ArtinM has been studied *in vitro* by stimulating isolated cell populations with the lectin ArtinM and monitoring the triggered responses; *in vivo*, the systemic and local effects of the ArtinM administration have been examined in mice infected with intracellular pathogens [7, 8, 10, 13, 21–24]. Although extensive, the *in vivo* evaluation still has an important lacuna that concerns the possible effects of ArtinM on normal, *naïve* mice. In the present study, we evaluated the systemic effects of different doses of ArtinM administration to *naïve* BALB/c mice. We found that ArtinM at high doses is associated with the occurrence of mild inflammatory infiltrates in tissues, neutrophil infiltration in the heart and lung, discrete alteration of serum biochemical parameters, and reduction of the IL-10 levels in tissues. Notably, ArtinM administration at the appropriate dose to induce immunomodulation only increased the frequency of CD4^+^ T cells slightly in the spleen and promoted no undesirable effects in the *naïve* mice.

## Materials and methods

### Animals

Male BALB/c mice at 6–8-weeks-old were acquired from the animal house of the Campus of Ribeirão Preto, University of São Paulo, Ribeirão Preto, São Paulo, Brazil. They were maintained under standard conditions in the animal house in the Molecular and Cellular Biology Department of the Faculty of Medicine of Ribeirão Preto, University of São Paulo, under optimized hygienic conditions. All experiments were conducted following the Committee of Ethics in Animal Research of the College of Medicine of Ribeirão Preto at the University of São Paulo approved the animal studies, Protocol no. 082/2012.

### ArtinM affinity purification

The lectin ArtinM was purified from the saline extract of *A*. *heterophyllus* (jackfruit) seeds via affinity chromatography on sugar columns as previously described [17]. The purity degree was evaluated by electrophoresis in a polyacrylamide gel (12%) in the presence of sodium dodecyl sulfate.

### Experimental design

**Fig 1:**
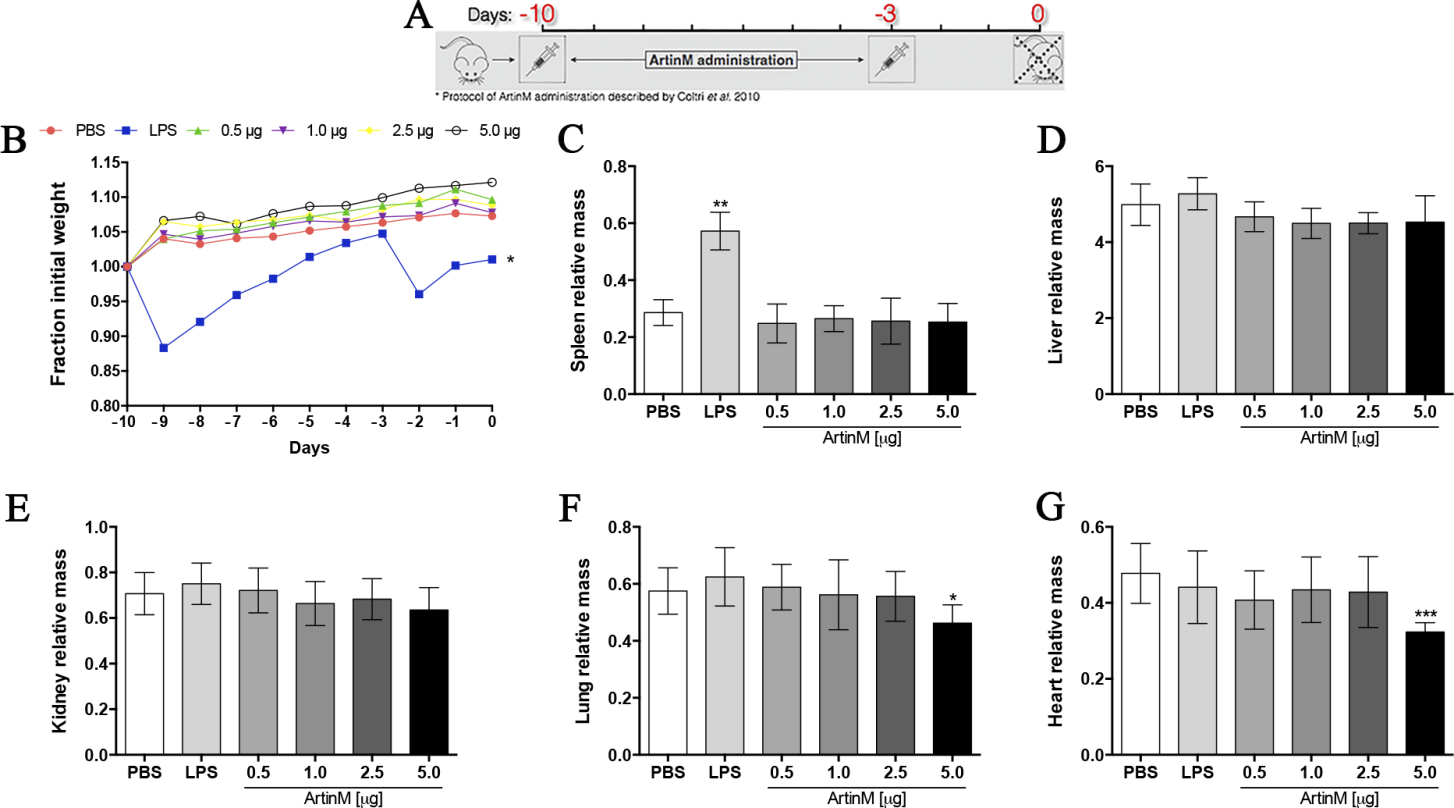
Body weight variation and relative organs mass of *naïve* BALB/c mice after ArtinM administration. (**A**) The protocol of ArtinM administration includes two subcutaneous injections, 10 and 3 days before the mice were euthanized (adapted from Coltri et al. [22]). An identical protocol was used for PBS or LPS injection in the negative and positive control groups, respectively. (**B**) The body weight was first determined on day -10, and the daily subsequent determinations allowed calculating the weight variations in relation to the first value. (**C–G**) On day 0, the mass of spleen (**C**), liver (**D**), kidney (**E**), lung (**F**), and heart (**G**) was measured, and the quotient between the organ mass and the body weight was used to express the organ relative mass (mg/body weight) for each mice. (**A**–**G**) Results are expressed as mean ± SD, and the differences were considered significant when p < 0.05 (*) compared to the PBS control group.

The administration protocol of ArtinM described by Coltri [21] was adapted for the current work. Groups of animals received two injections subcutaneously (s.c.; at 7-day intervals) of ArtinM, LPS, or PBS, as indicated in Fig 1. The injected ArtinM doses were 0.5, 1.0, 2.5, or 5.0 μg. Mice of the positive control group received LPS (3.2 mg/Kg), whereas those of the negative control group received PBS. Mice were euthanized at day 0 to collect blood and organs.

### Measurement of variation in body weight and relative mass of organs

Body weight of the animals was measured daily to calculate the variation from the initial body weight and was expressed as a percentage of the initial body weight. The quotient of organ mass and the body weight was determined at day 0 to estimate the relative mass of the organs (spleen, heart, lung, liver, and kidney).

### Histopathological processing and morphometric analysis

A fragment of either of the organs (heart, lung, liver, and kidney) was processed for histopathological and morphometric analysis. In brief, specimens were fixed in methacarn, dehydrated in a series of ethyl alcohol, diaphanized in xylol, and embedded in paraffin. Serial sections with a thickness of 6 μm were prepared at intervals of 30 μm. Tissues were stained with hematoxylin and eosin (H&E), and 20 samples were analyzed for each organ.

Morphometric analysis was performed on one slide (randomly selected) for all organs. Four images were captured per slide, selecting those obtained at 60-μm intervals. The analysis was performed using a light microscope fitted with a digital camera (Evolution MP 5.0; Media Cibernetic Inc., USA) and using the Image-Pro Plus software (Media Cibernetic Inc., USA). Images of the histological sections were captured using 20× objective lenses and were analyzed by the ImageJ software in duplicates.

Each acquired image was subdivided into 25 grids (155,2015 μm^2^ each), and 13 grids were randomly selected to be analyzed. The inflammatory infiltrates were examined in a total area of 1,614 mm^2^ per organ/animal. The quantitation of the number of inflammatory cells (inflammatory infiltrate/cm^2^) in each tissue was calculated as follows: (inflammatory cells)/(total area).

### Peripheral blood leukogram

The peripheral blood samples of each animal were used to measure the total leukocyte count by Neubauer chamber after 1:20 dilution in Turkey’s solution. Next, peripheral blood samples were smeared on a glass slide and stained with Panopticon (NewProv, Products for Laboratory LTDA, Brazil) to perform differential leucocyte count. Neutrophils, lymphocytes, and monocytes were counted using a light microscope under a 100× objective lens.

### Measurement of biochemical markers in serum

The levels of urea, glutamic oxaloacetic transaminase (GOT), glutamic pyruvic transaminase (GPT), creatine phosphokinase (CPK), creatine kinase MB isoenzyme (CK-MB), alkaline phosphatase, protein total, albumin, and globulin were determined in plasma samples by using kits obtained from ROCHE (Roche Diagnostics Ltd), according the manufacturer’s instructions. The concentration of biochemical markers was determined from standard curves in the Cobas^®^ Integra 400 equipment (Cobas Integra, Roche Diagnostics Ltd).

### Measurement of cytokines

The lung, liver, heart, spleen, and kidney were homogenized, centrifugated (3220 ×g for 10 min at 4°C), and the supernatants were assessed to determine the levels of IL-12p40, IL-10, IFN-γ, and TNF-α by capture enzyme-linked immunosorbent assay (ELISA) with antibody pairs purchased from BD Biosciences (Pharmingen, San Diego, CA, USA), according to the manufacturer’s protocol. The cytokines concentrations were determined concerning a standard curve for each murine recombinant cytokine. The data represent the mean of three independent assays.

### Myeloperoxidase (MPO) activity assay

Fragments of lung, liver, heart, kidney, and spleen were individually homogenized in phosphate sodium buffer (50 mM; pH 7.5) containing 0.5% hexadecyltrimethylammonium bromide (HTAB; 50 mM) and were stocked at −80°C for 24 h. Then, the samples were centrifuged (3000 ×g for 30 min at 4°C). The supernatant was distributed in 96-well microplates and 50 μL of O-dianisidine (3.51 mg of O-dionisidine in 5 mL of phosphate buffer and 5 μL of H_2_O_2_ at 30%) was added. The plate was incubated at 37°C for 30 min, and the reaction was stopped by adding sodium azide (1.0%). Absorbance was recorded at 460 nm using a Power Wave-X microplate scanning spectrophotometer (BioTek Instruments, Inc., Winooski, VT, USA). The quotient between the optical density value and the organ weight was calculated to express the MPO activity.

### Flow cytometry analysis of spleen cells and pulmonary leukocytes

The spleen of each mouse was removed aseptically and transferred to a petri dish, soaked, macerated, and filtered using a nylon strainer (40 μm) by using RPMI medium. The cellular suspension was centrifuged (300 ×g for 10 min at 4°C), and erythrocytes were depleted with lysis buffer (9 parts 0.16 M ammonium chloride and 1 part 0.17 M Tris–HCl, pH 7.5) for 5 min in an ice bath. Then, the cells were fixed in PBS–formaldehyde (3%), washed with PBS–glycine (1%), and with PBS for removing formaldehyde. The number of cells contained in the suspensions was determined, and 5 × 10^5^ cells were incubated with anti-CD4 FITC (clone H129.19), anti-CD8 FITC (clone 53–6.7), anti-CD3 PE (clone 145-2C11), anti-CD11b PE (clone M1/70), anti-CD19 PE (clone 1D3), or IgG Isotype control antibody for 45 min at 4°C. After washing twice with PBS, the cells were analyzed by flow cytometry (Guava easyCyte, Guava Technologies, Millipore, Hayward, CA, USA).

The lung tissues were excised on day 0 and subjected to enzymatic digestion at 37°C for 30 minutes in 1 ml of RPMI medium containing 1 mg/ml of collagenase type IV. After, the tissues were dissociated in a 40-μm nylon cell strainer (BD Biosciences, San Diego, CA, USA) and centrifuged at 300g for 10 min at 4°C with RPMI supplemented with 10% fetal cow serum (FCS). The suspension was erythrocyte-depleted with lysing buffer (9 parts 0.16 M ammonium chloride and one part 0.17 M Tris–HCl, pH 7.5) for 10 min at 4°C. Afterwards, the pulmonary leucocytes were washed in PBS and the cell concentration was determinated. The cells were stained with anti-CD4 FITC (clone H129.19), and anti-CD3 PE (clone 145-2C11) antibodies or IgG Isotype control antibody at 4°C. After 45 min, the cells were washed in PBS and analyzed by flow cytometry (FACSCalibur, Biosciences, CA, USA).

### Quantitative reverse transcription (qRT)-PCR of transcription factors

Total RNA was isolated from spleen cells using the TRIzol Reagent, according to the manufacturer’s instructions. Reverse transcription of RNA into cDNA was performed using the ImProm-II Reverse Transcription System (Promega, Fitchburg, WI) using oligo(dT). qRT-PCR was performed in 15-μL reaction mixtures with SYBR Green (Applied Biosystems/Life Technologies, Carlsbad, CA, USA). The reactions were performed using the 7500 Real-Time PCR System (Applied Biosystems) under the following conditions: 50°C for 2 min, 95°C for 10 min, and 40 cycles of 95°C for 15 sec/60°C for 1 min. Gene expression was quantified using the ΔΔCt method and normalized to β-actin expression. The following PCR primers were utilized: β-actin (F: 5′-AGCTGCGTTTTACACCCTTT-3′/R: 5′-AAGCCATGCCAATGTTGTCT-3′); T-bet (F: CACTAAGCAAGGACGGCGAA/R: CCACCAAGACCACATCCAC); GATA-3 (F: AAGAAAGGCA TGAAGGACGC/R: GTGTGCCCA TTTGGACA TCA); ROR-γt (F: TGGAAGATGTGGACTTCGTT/R: TGGTTCCCCAAGTTCAGGAT).

### Statistical analysis

Data were analyzed using Graph Pad Prism 6.0 (GraphPad Software, Inc. La Jolla, CA., USA), and the results are expressed as mean ± standard deviation (SD). All statistical determinations for normality were analyzed by the Kolmogorov–Smirnov test, and homogeneous variance was determined by the Bartlett’s test. The difference between means of groups was performed with analysis of variance for one-way ANOVA test followed by Bonferroni’s multiple comparison tests. Differences with p < 0.05 were considered statistically significant.

## Results

### Effects of ArtinM administration on body and organ weights of *naïve* BALB/c mice

ArtinM-induced Th1 immunity provides protection against several fungal and protozoan infections [8–10, 21–24]. Thus, evaluating the effects of ArtinM on *naïve* mice was mandatory. We adopted a previously standardized protocol [22], and administered ArtinM according to the schedule shown in Fig 1A. Each group of *naïve* mice received low (0.5 and 1.0 μg) or high (2.5 and 5.0 μg) doses of ArtinM. For the entire experimental period, we daily measured the body weight of animals, and analyzed its variation relative to the initial verification (day -10). The constructed time-lapse curves showed that no significant change in body weight was observed in the ArtinM-injected mice compared to that in the negative control group, which received PBS instead of the lectin (Fig 1B). Moreover, mice injected with LPS (positive control) exhibited body weight loss a day after each endotoxin injection (Fig 1B). Also, at the end of the experimental period (day 0), we measured the weight of the harvested organs (spleen, liver, kidneys, lungs, and heart) and determined the quotients between weight of each organ and body weight at day 0. The relative mass of each organ of mice receiving lower ArtinM doses (0.5–1.0 μg) was similar to that verified in the PBS control group (Fig 1C–1G). Nonetheless, in the group receiving the ArtinM highest dose (5.0 μg), we detected a significantly lower relative mass of the heart and lung than that verified in the PBS control group (Fig 1F and 1G). These results showed that ArtinM administered at the appropriate dose to induce immunomodulation did not promote body weight loss nor did it alter the mass of the organs. However, ArtinM administration at the highest dose reduced the relative mass of the heart and lung.

### Evaluation of inflammatory infiltrate in tissues of *naïve* BALB/c after ArtinM administration

**Fig 2:**
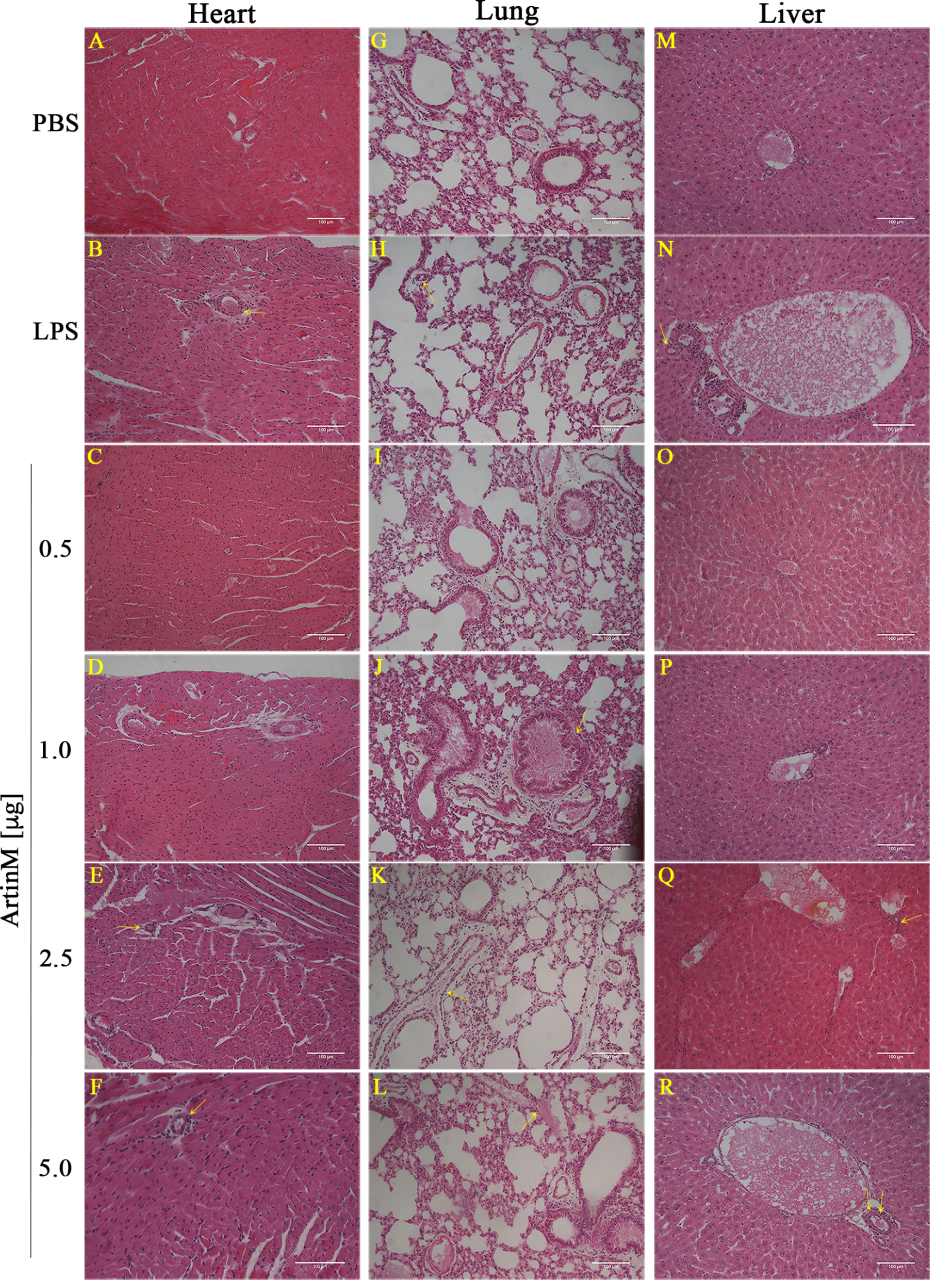
Histopathology of the heart, lung, and liver of *naïve* BALB/c mice receiving ArtinM. The panels show representative sections (6 μm) of the organs from mice injected with various ArtinM doses or those of the positive (LPS) and negative (PBS) controls groups. The sections were stained with hematoxylin and eosin (H&E), and images were captured using a microscope (Nikon Eclipse 50i) coupled to a digital camera (Evolution MP 5.0). The yellow arrows indicate the presence of inflammatory infiltrate at the perivascular, peribronchial, or periductal regions. Magnification bars = 100 μm for all the tissues sections.

**Fig 3:**
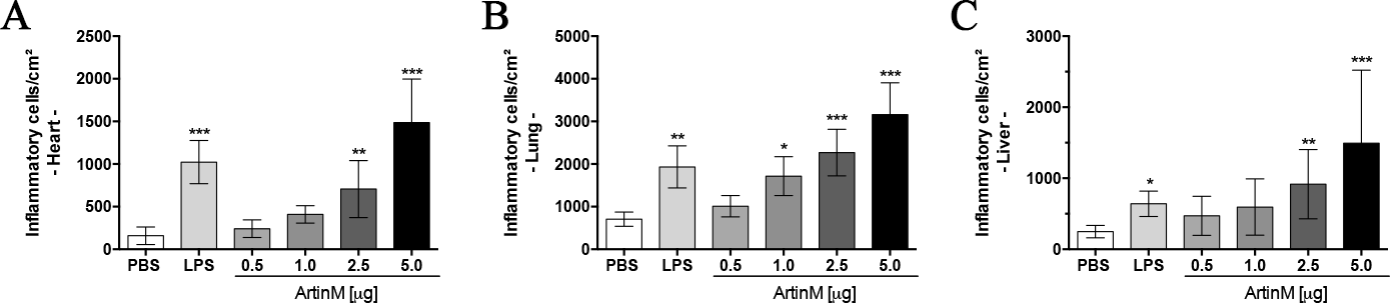
Quantitation of inflammatory infiltrate in the heart, lung, and liver of *naïve* BALB/c mice receiving ArtinM. The inflammatory infiltrate were quantitated in captured images of two tissue sections of 6 μm, obtained at an interval of 60 μm for each organ stained with H&E. The morphometric analysis was performed using the ImageJ software, as described in Material and Methods section. Tissue samples were obtained from mice injected with various ArtinM doses, LPS (positive controls), or PBS (negative controls). Inflammatory cells were counted and represented as number of inflammatory cells/cm^2^. The results are expressed as mean ± SD, and the differences were considered significant when p < 0.05 (*) compared to the PBS control group.

The alteration in organ mass associated with high-dose ArtinM administration propelled us to perform histopathological analysis of the heart, lung, kidney, and liver after the lectin injection. These tissues, stained with H&E, exhibited morphology comparable to that of the control group. Few and discrete perivascular foci of mononuclear inflammatory cells were observed in the heart, lung, and liver of mice receiving high doses of ArtinM or LPS (Fig 2). Moreover, the same group of animals displayed few mononuclear inflammatory cells at the peribronchial and periductal regions of the lung and liver, respectively (Fig 2J, 2K, 2Q and 2R); we visualized no inflammatory infiltrate in the kidney of any experimental group (S1 Fig). A morphometric semiquantitative analysis of the inflammatory foci in the heart, lung, and liver showed that the organs of the mice injected with high doses of ArtinM (2.5 and 5.0 μg) showed higher numbers of inflammatory foci compared to those observed in the PBS control group (Fig 3). Moreover, we verified by flow cytometry a significant increase in the CD4^+^ T cells frequency among pulmonary leukocytes harvested from mice that received high doses of ArtinM (S2 Fig). These findings suggest that ArtinM administration at high doses is associated with the occurrence of inflammatory infiltrates in tissues of *naïve* mice.

### Serum biochemistry of *naïve* BALB/c mice after ArtinM administration

**Fig 4:**
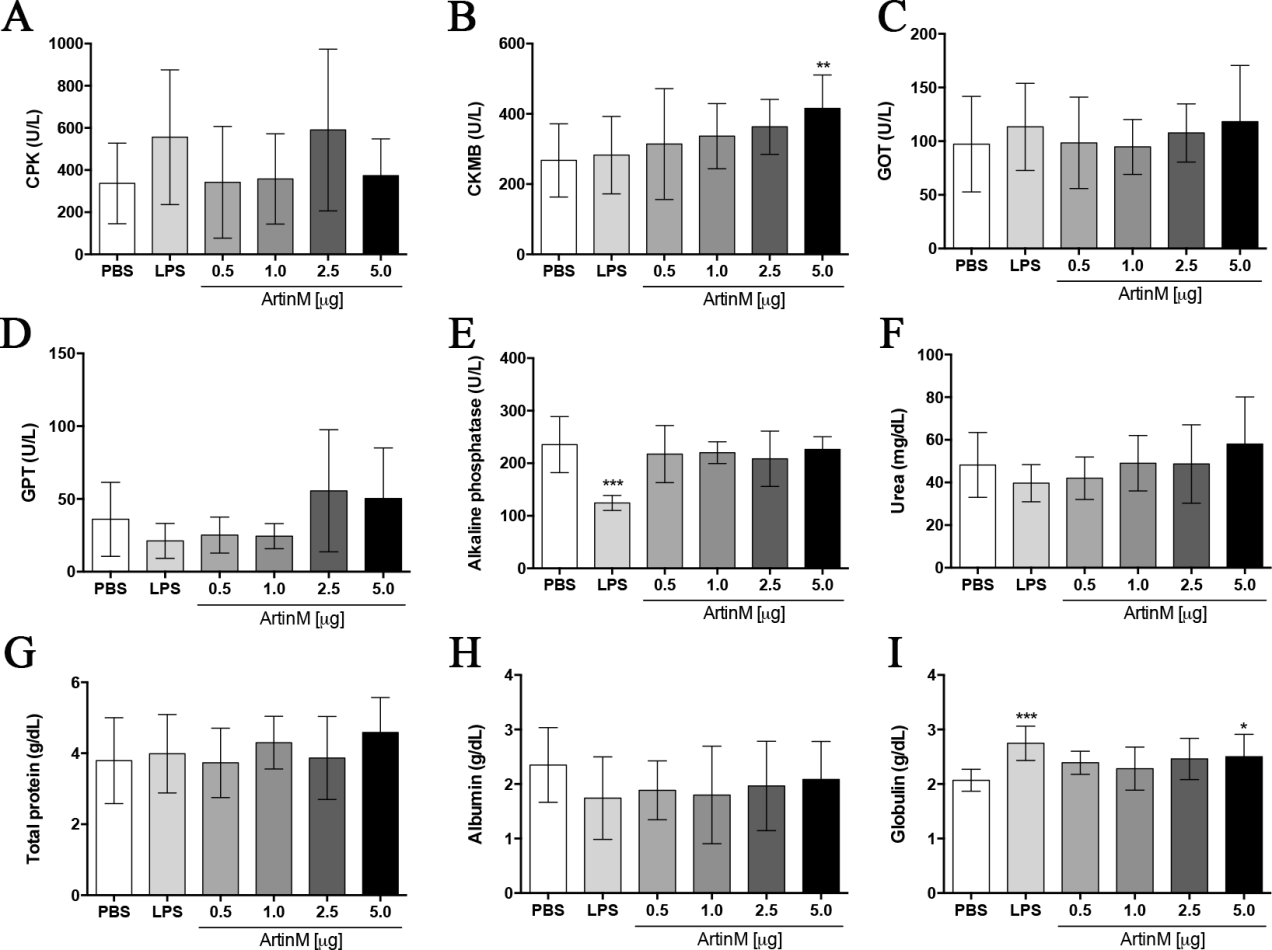
Serum biochemical parameters of *naïve* BALB/c mice receiving ArtinM. The serum levels of CPK (**A**), CK-MB (**B**), GOT (**C**), GPT (**D**), alkaline phosphatase (**E**), urea (**F**), total protein (**G**), albumin (**H**), and globulin (**I**) were determined in samples harvested at day 0 from mice that received ArtinM at the specified dose (x axis). PBS and LPS was administered to the negative and positive controls, respectively. Results are expressed as mean ± SD, and differences were considered significant when p < 0.05 (*) compared to the PBS control group.

The observation that some organs of mice receiving high doses of ArtinM present with reduced mass and mild inflammatory infiltrates directed us to examine various serum biochemical parameters. We compared the levels of urea, GOT, GPT, CPK, CK-MB, alkaline phosphatase, protein total, albumin, and globulin in animals treated with or without ArtinM. Although most parameters were similar in mice that received ArtinM or PBS (Fig 4), we found a significant increase in the levels of CK-MB and globulin in animals receiving the highest dose of ArtinM (5.0 μg; Fig 4B and 4I). These results reinforced the idea that ArtinM at high dose may exert a systemic effect in *naïve* mice.

### Effect of ArtinM administration on the peripheral blood leukogram and levels of tissue MPO

**Fig 5:**
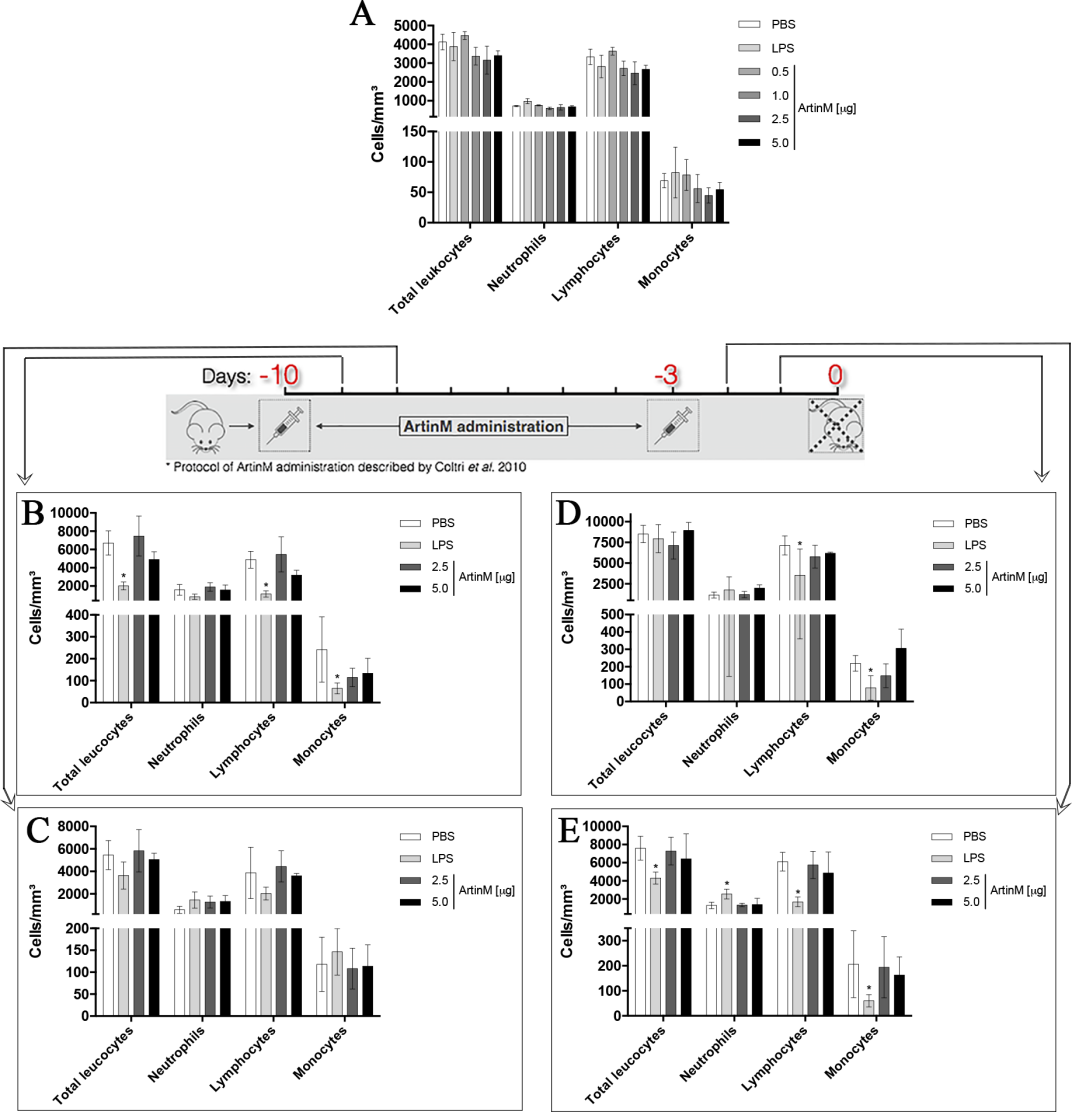
Blood leukogram of *naïve* BALB/c mice after ArtinM administration. Total and differential leukocyte counting was performed in blood samples obtained at days 0 (**A**), -9 (**B**), -8 (**C**), -2 (**D**), and -1 (**E**) from animals that received ArtinM at the doses specified in each panel, LPS (positive controls), or PBS (negative controls). Results are expressed as mean ± SEM (**A**) and mean ± SD (**B-E**). Differences were considered significant when p < 0.05 (*) compared to the PBS control group.

We evaluated the leukogram of the samples collected at the end of the experimental period (day 0). We found that ArtinM injection at low and high doses, compared to the PBS injection, caused no alteration in the number of neutrophils, lymphocytes, and monocytes (Fig 5A). We also determined the leukogram at subsequent days after ArtinM administration at high doses, and no differences were observed in comparison to the PBS control groups (Fig 5B–5E). In contrast, LPS administration significantly reduced the number of lymphocytes and monocytes compared to the PBS control group (Fig 5B–5E).

**Fig 6:**
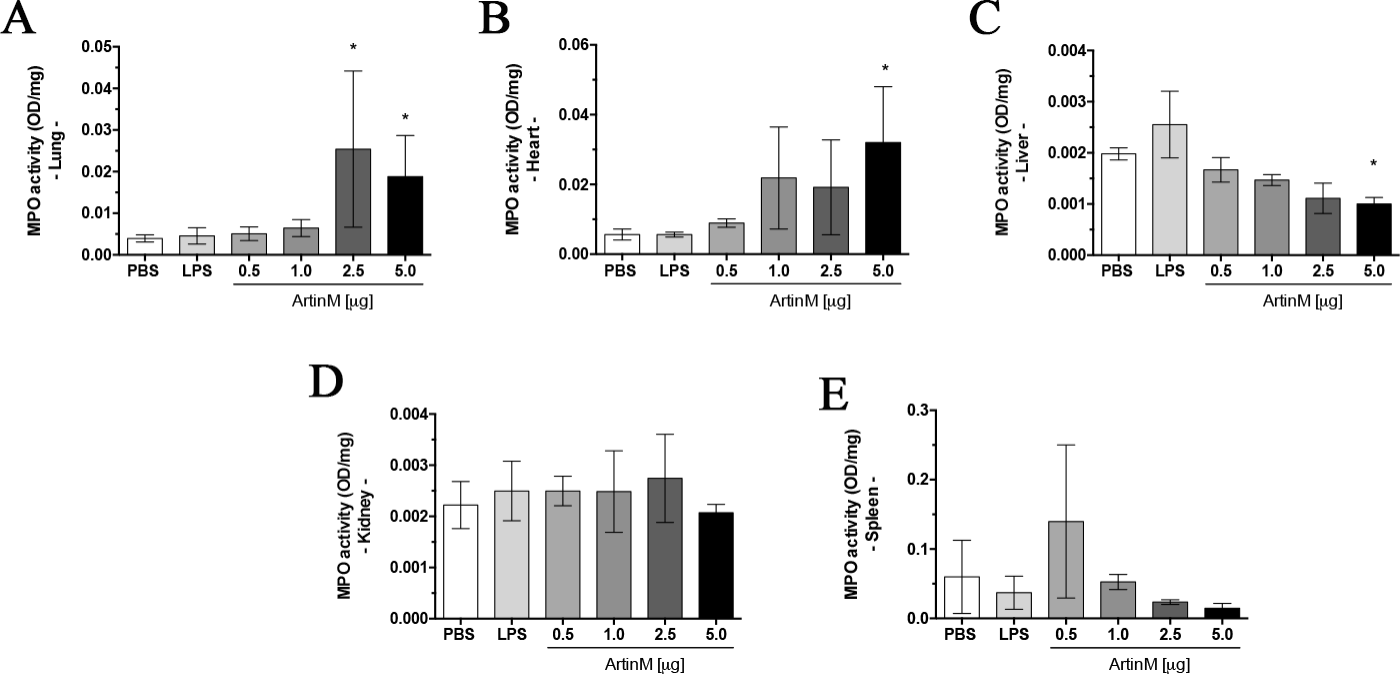
MPO activity in organs of *naïve* BALB/c mice after ArtinM administration. The supernatant of the homogenates of organs obtained at day 0 was incubated with O-dianisidine at 37°C for 30 min to measure the MPO activity, which was represented as optical density (OD)/organ mass (mg). The organs were lung (**A**), heart (**B**), liver (**C**), kidney (**D**), and spleen (**E**). Animals received ArtinM at the specified doses, PBS (negative control), or LPS (positive control). Results are expressed as mean ± SD, and the differences were considered significant when p < 0.05 (*) compared to the PBS control group.

Considering that ArtinM activity induces neutrophil migration and activation, we examined mice organs for the presence of neutrophils, indicated by the grade of MPO activity detected in tissues. The lung, heart, kidney, spleen, and liver were harvested at the experimental period terminus (day 0), and we used the supernatant of the organ homogenate to measure the MPO activity. We found that administration of ArtinM at the highest dose was associated with a significant increase in MPO activity in the lung and heart compared to that observed in the PBS control group (Fig 6A and 6B). However, MPO activity was reduced in the liver of animals injected with 5.0 μg of ArtinM (Fig 6C). Our results indicated that ArtinM administration at the highest dose augments the neutrophil extravasation to tissues of *naïve* mice.

### Tissue levels of pro- and anti-inflammatory cytokines in *naïve* BALB/c mice after ArtinM administration

**Fig 7:**
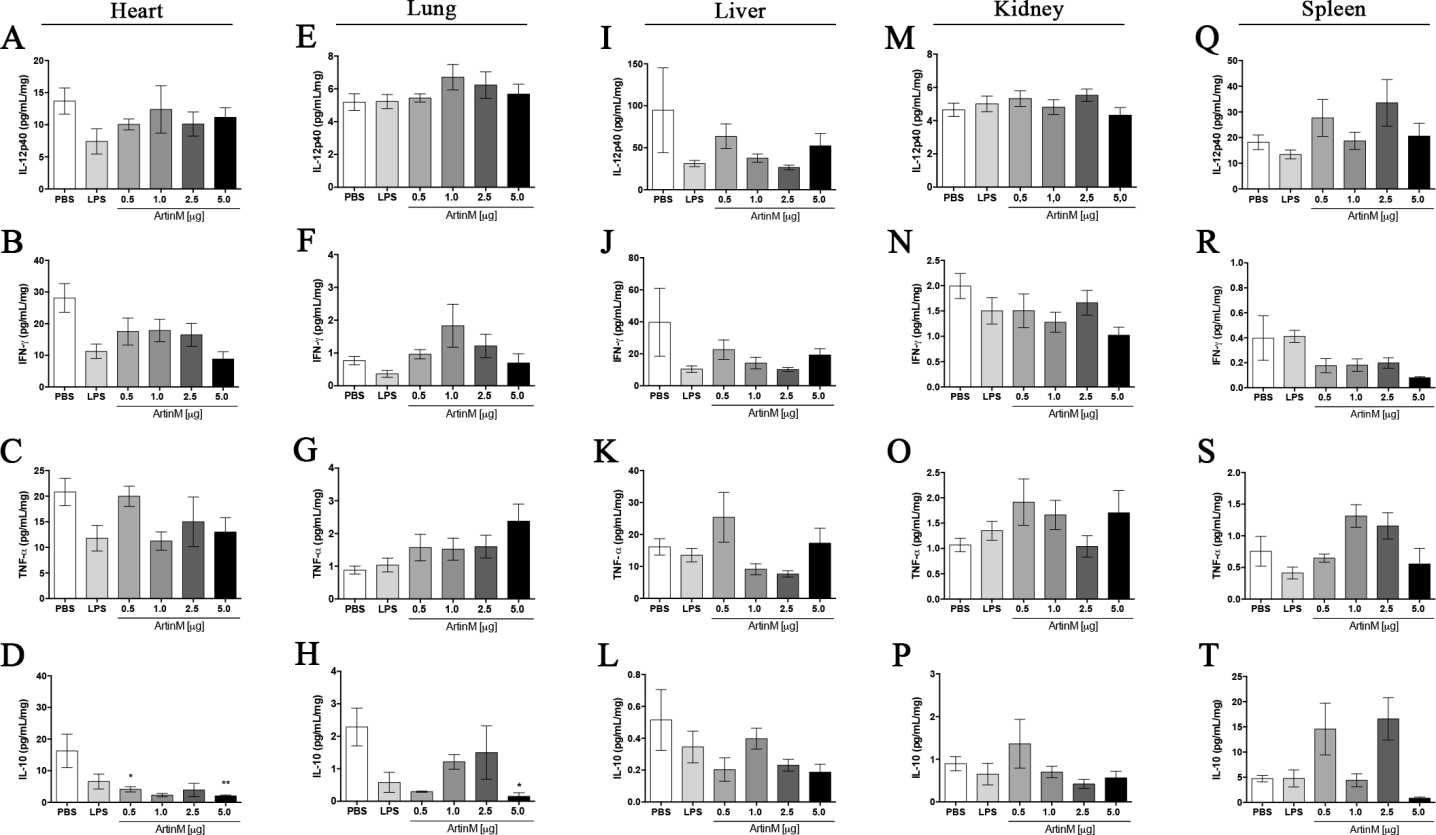
Cytokine levels in tissues of *naïve* BALB/c mice after ArtinM administration. The levels of IL-12p40, IFN-γ, TNF-α, and IL-10 in the supernatant of organ homogenates obtained at the day 0 were assessed by ELISA. The organs were heart (**A**–**D**), lung (**E**–**H**), liver (**I**–**L**), kidney (**M**–**P**), and spleen (**Q**–**T**). Animals received ArtinM at the specified doses, PBS (negative control), or LPS (positive control). Results are expressed as mean ± SD, and the differences were considered significant when p < 0.05 (*) compared to the PBS control group.

Previous studies have demonstrated that the prophylactic or therapeutic effect of ArtinM against several pathogens is exerted through the induction of a prominent production of pro-inflammatory cytokines [8, 21, 22]. In the current study, we verified that ArtinM at high doses induces mild tissue infiltration of inflammatory cells. Then, we compared the IL-12, IFN-γ, IL-10, and TNF-α levels in homogenates of organs from ArtinM-, PBS-, and LPS-injected mice. No significant difference was observed among the groups regarding the concentration of cytokines observed in the liver, kidney, and spleen (Fig 7I–7T). Interestingly, lower IL-10 levels were detected in the heart and lung of mice receiving 5.0 μg of ArtinM compared to that observed in the PBS control group, whereas no difference was observed in the IL-12, IFN-γ, IL-10, and TNF-α levels (Fig 7D and 7H). Thus, the only substantial alteration was observed in IL-10 levels, which were reduced in the heart and lung tissues after the ArtinM administration at the highest dose.

### Phenotype of the spleen cells of ArtinM-injected *naïve* mice

**Fig 8:**
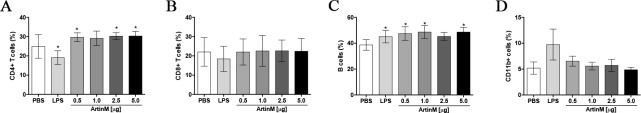
Relative frequency of cell populations in the spleen of *naïve* BALB/c mice after ArtinM administration. Cell suspension prepared from the mice spleen harvested at day 0 was assessed by flow cytometry. The frequency of CD4+ T (**A**), CD8+ T (**B**), B (**C**), and CD11b+ cells (**D**) was determined by reacting the cells with anti-CD4 FITC, anti-CD8 FITC, anti-CD3 PE, anti-CD19 PE, and anti-CD11b PE antibodies. Animals received ArtinM at the specified doses, PBS (negative control), or LPS (positive control). Results are expressed in percentage and are represented as mean ± SD. Differences were considered significant when p < 0.05 (*) compared to the PBS control group.

To investigate whether or not ArtinM administration alters the incidence of immune cell populations, we prepared suspensions of spleen cells that were harvested at day 0 from mice of the experimental and control groups. The cell suspensions were analyzed by flow cytometry to determine the relative frequency of the major cell populations in the spleen. We found a significant increase in CD4^+^ T and B cells in mice receiving different ArtinM doses compared to that observed in the PBS control group (Fig 8A and 8C). In contrast, the frequency of CD8^+^ T and CD11b cells in the spleen of ArtinM-injected mice was similar to that observed in the PBS control group (Fig 8B and 8D). Our results indicate that ArtinM administration augments the frequency of Th and B cells in the spleen of *naïve* mice.

**Fig 9:**
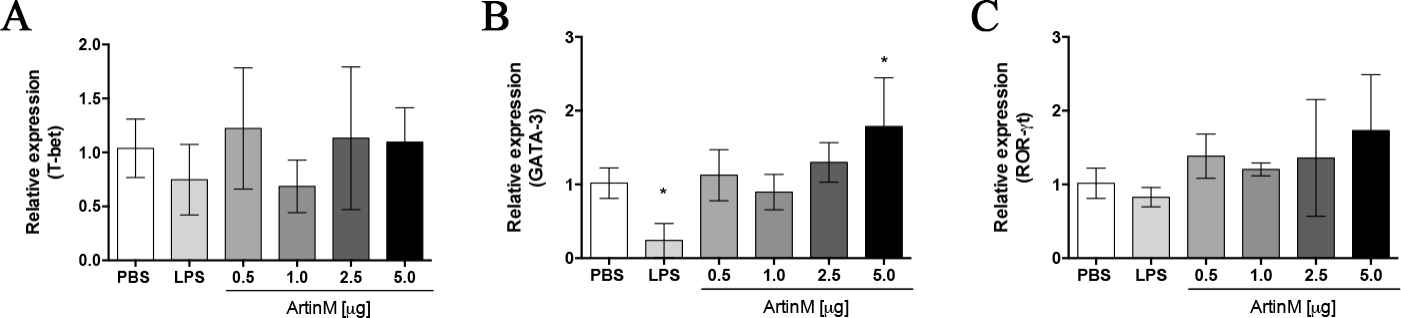
Relative expression of transcription factors related to the differentiation of T helper cells in the spleen of *naïve* BALB/c mice after ArtinM administration. Total RNA was extracted from the mice spleen harvested at day 0. The total RNA was reverse-transcribed into cDNA, and the relative expression of T-bet (**A**), GATA-3 (**B**), and ROR-γt (**C**) was determined by real-time quantitative PCR. Animals received ArtinM at the specified doses, PBS (negative control), or LPS (positive control). The values were normalized to β-actin expression. Results are expressed as mean ± SD, and the levels of relative expression were compared to the PBS control group. Differences were considered significant when p < 0.05 (*) compared to the PBS control group.

The higher frequency of CD4^+^ T cells in the spleen of mice receiving ArtinM propelled us to assess the relative expression of transcription factors involved in differentiation of Th cells, namely T-bet (Th1 cells), GATA-3 (Th2 cells), and ROR-γt (Th17 cells), by RT-PCR. The only significant difference observed between ArtinM- and PBS-injected groups was the augmented GATA-3 expression in animals receiving the highest ArtinM dose (5.0 μg; Fig 9C). However, administration of ArtinM, even at high doses, did not modify the relative expression of T-bet and ROR-γt (Fig 9A and 9B) in spleen cells. These results suggest that the augmented frequency of CD4+ T cells detected in the spleen of ArtinM-injected *naïve* mice was not associated with changes in the relative expression of transcription factors associated with T cell differentiation.

## Discussion

The immunomodulatory activity of ArtinM depends on the recognition of TLR2 N-glycans, which drives the immunity toward the Th1 axis and confers protection against several intracellular pathogens. Considering the potential applicability of ArtinM as a therapeutic agent and the consequent necessity of evaluating its collateral effects, we investigated the systemic effects of ArtinM administration at different doses in *naïve* BALB/c mice. We observed that high doses of ArtinM induced augmentation of the serum levels of CK-MB and globulin, inflammatory infiltrates in several tissues, and increased MPO and IL-10 levels in the heart and lungs. However, *naïve* BALB/c mice receiving low doses of ArtinM displayed increased frequency of CD4+ T and B cells in the spleen. Low doses of ArtinM that are therapeutic against experimental infections [8, 10, 21–24] did not exert any deleterious effects in healthy mice, in contrast with the systemic consequences of ArtinM injection at higher doses.

The adopted protocol of ArtinM administration, shown in Fig 1A, was designed on the basis of previous standardization [8, 10, 21, 22, 24, 32, 33] that was demonstrated to confer resistance against several intracellular pathogens [8, 10, 21–24].

The s.c. route of protein injection, compared to oral administration, affords the advantage of preventing the molecule from losing its stability due to hydrolysis occurring in the protein passage through the gastrointestinal tract and favoring protein bioavailability [34]. Previous studies have shown that proteins with a high isoelectric point (pI) [34, 35] exhibit a positive correlation between their molecular mass (MM) and half-life permanence at the injection site [34, 36]. Molecules that are injected s.c. may be transported through the blood and/or lymphatic vessels in a manner that is dependent on the MM. The blood capillaries preferentially distribute molecules with MM lower than 1 kDa [37], whereas the lymphatic system drains molecules with MM >16 kDa [36–40]. ArtinM is a homotetramer consisting of 16-kDa subunits [17], a feature that allows us to predict that s.c. injected ArtinM is captured and transported by the lymphatic system. Since that ArtinM was injected in the groin, a region that is enriched with lymph nodes, its presence in the secondary lymphoid tissue could induce a specific adaptive response. Nonetheless, s.c. injected ArtinM at a therapeutic dose, even if associated with a Freund’s adjuvant, does not trigger a detectable specific immune response (Coltri et al., unpublished data). However, in infected mice, in which the sensitized macrophages and dendritic cells display increased TLR expression in response to the pathogen stimulus, ArtinM elicits additional cell activation through the recognition of TLR2 N-glycans, generation of M1 macrophages, and induction of Th1 responses [8, 21, 23] (da Silva et al. unpublished data). Thus, we found that s.c. injected ArtinM does not alter TLR2 expression in the spleen cell suspension compared to that in the PBS control group (S3 Fig), although macrophages under ArtinM stimulus significantly increase TLR2 expression [41]. Regarding that we did not find an altered cytokine profile in *naïve* BALB/c mice, we assumed that ArtinM administered at a therapeutic dose does not influence the immune system of the animals. After passing through the lymph nodes, s.c. injected ArtinM enters the thoracic lymph duct and reaches the blood vessels allowing systemic distribution of the proteins [40, 42]. ArtinM capture by the lymphatic system makes possible the lectin to pass through the appropriate circuit for exerting its immunomodulatory effect, while preserving its bioavailability as an immunomodulatory agent and reproducing advantages that were already reported for vaccines and adjuvants [36, 42, 43]. Recently, Broad et al. demonstrated that mice maintained in an enriched environment (EE) favors the modulation of their immune response, determined by infecting the mice and verifying that the inflammatory response favors the resolution of the systemic infection [44]. The immunomodulation induced by EE and ArtinM are similar because both are imperceptible in *naïve* mice but notable in infected animals [44].

In our experiments, the s.c. injection of LPS was used as the positive control; its systemic effects such as the reduction in weight body occurred within 24 h after administration, as previously reported in *naïve* C57BL/6 mice [45, 46]. Similarly, in the present study, we observed a reduction in body weight of *naïve* BALB/c mice 96 h after s.c. injecting LPS. These findings demonstrate that s.c. injected LPS presents high bioavailability, which is probably related to its MM, and could be changed by aggregating the molecule [34, 36]. Body weight of the mice receiving ArtinM at different doses did not alter compared to the body weight of mice in the PBS control group. However, this observation cannot be attributed to a low bioavailability of ArtinM since the positive control was active and other systemic effects of ArtinM at high doses were manifested, such as change in organ weight and the presence of tissue inflammatory infiltrates.

Histopathological analysis of different mice organs allows detection of the systemic effects of an immunomodulatory agent administration to *naïve* mice. However, this approach has not been executed previously to evaluate the effect of an agent that recognizes N-glycans to interact with TLR2 and modulates the host immunity. In the current study, we performed histological analysis of the heart, kidney, lung, and liver of *naïve* BALB/c mice injected with ArtinM. Interestingly, we did not observe morphological alterations in the tissues from *naïve* animals that received a low ArtinM dose (0.5 μg, dosage administered to prevent or treat infections) compared to those in the tissues from the PBS control group. Otherwise, administration of ArtinM at high doses was associated with the presence of discrete foci of mononuclear cells infiltration in the heart, lung, and liver. The infiltration was prominent in the perivascular, peribronchial, and periductal areas, indicating that ArtinM administration at high doses has systemic effects in *naïve* BALB/c mice. These findings support the microscopic detection of discrete mononuclear cells infiltrations and show a high frequency of CD4^+^ T cells in the lung obtained from *naïve* mice that received high doses of ArtinM (S2 Fig). Our observations reinforce our postulation that s.c. administration of ArtinM is appropriate for exerting its systemic effects, likely facilitated by the protein passage throughout the cardiac and pulmonary circulation [34, 36–40, 42]. We suppose that the inflammatory infiltrate verified in the heart, lung, and liver of *naïve* BALB/c mice treated with high ArtinM doses may also occur in the unexamined organs of these animals.

The evaluation of serum biochemical parameters revealed additional systemic effects of ArtinM administration at high doses to *naïve* BALB/c mice, concerning the increased serum levels of globulins and CK-MB. Previous study reported that ArtinM induces polyclonal B cell activation, followed by high immunoglobulins secretion, in a manner that is T cell-independent [47]. Recently, our group demonstrated that ArtinM induces the activation of murine CD4^+^ and CD8^+^ T cells [13, 14, 48], whereas in the present work we found that ArtinM treated mice exhibit a significant increase in the frequency of CD4^+^ T and B cells in the spleen (Fig 8). The ability of ArtinM to elicit the activation of adaptive immune cells [13, 14, 48] seems to be related to augmented production of immunoglobulins and the resultant alteration in the serum proteins. The augmented levels of CK-MB that follow the ArtinM administration at high doses may be associated with the presence of mononuclear cells and high levels of MPO in the heart. It is well established that MPO has pleiotropic actions, directly linked with the interference of MPO-derived oxidants with numerous cell functions, contributing to tissue injury in a wide spectrum of diseases. Nonetheless, the most prominent MPO effect is exerted on the initiation and the propagation of a number of cardiac pathologies [49, 50]. It was demonstrated that high levels of MPO can generate a cytotoxic effect in cardiac tissue [49–51]. Moreover, the early MPO releasing does not require the existence of a previous morphological alteration [49]. Thus, we postulate that s.c. administered ArtinM is systemically bioavailable. The low levels of FAL verified in *naïve* BALB/c mice after LPS administration are probably due to the ability of FAL to dephosphorylate and inactivate LPS. The catalytic activity and enzymatic turnover that follow FAL–LPS interaction reduce the FAL levels [52–54].

Considering that ArtinM activates innate and adaptive immune cells [8, 13–15, 17, 55–58], we performed leukograms of blood samples harvested from *naïve* BALB/c mice treated with different doses of ArtinM. Samples collected at 24 and 48 h after the s.c. injections of ArtinM provided leukograms that did not differ from those of the PBS control mice, whereas s.c. injected LPS promoted a significant decrease in the total leukocyte number within 24 h. Interestingly, the measurement of MPO activity revealed a significantly augmented number of neutrophils in the heart and lungs of the *naïve* BALB/c mice receiving ArtinM at high doses. These findings are corroborated by previous studies that observed the ArtinM capacity to induce the adhesion and haptotactic migration neutrophil in lung [16, 17, 56], and the proinflammatory activity of neutrophils is capable to exert a deleterious effect in the heart [45, 46].

The ability of ArtinM to modulate the immune response of infected BALB/c mice led us to characterize the profile of cytokines harvested from the organs of *naïve* mice receiving s.c. injections of ArtinM. Mice receiving ArtinM at high doses displayed reduced IL-10 content in the heart and lungs, which in addition to the increased MPO activity indicates a proinflammatory environment promoted by ArtinM at high doses. Concerning the phenotypic profile of spleen cells, we demonstrated a significant increase in the frequency of CD4+ T cells, which was not associated with higher incidences of Th and B cells, following s.c. injections of ArtinM at different doses. These findings corroborate with our hypothesis that the initial ArtinM distribution by lymphatic system favors the lectin to exert its effect on immune cells.

In conclusion, our study demonstrates that s.c. injection of ArtinM at high doses to *naïve* BALB/c mice induces a proinflammatory response that accounts for deleterious effects in the animals. In contrast, the administration of ArtinM at therapeutic doses, which were previously standardized in murine models of infections caused by various intracellular pathogens, does not exert a deleterious effect in *naïve* BALB/c mice. Moreover, s.c. administration of ArtinM is appropriate to preserve its bioavailability, which is desirable for its application as an immunomodulatory agent to confer resistance to murine models of human infectious diseases.

## Supporting information

S1 Fig Histopathology of the kidney of naïve BALB/c mice after ArtinM administration. The panels show representative images of kidney sections harvested at day 0 from mice receiving ArtinM at the specified doses, PBS (negative control), or LPS (positive control). The sections were stained with hematoxylin and eosin (H&E), and images were captured using a microscope (Nikon Eclipse 50i) coupled to a digital camera (Evolution MP 5.0). Magnification bars = 100 μm for all sections.
(TIF)

S2 Fig Relative frequency of CD4^+^ T cells in the lung of naïve BALB/c mice that were injected with ArtinM at high doses. Pulmonary leukocytes harvested from naive mice at day 0 following administration of ArtinM at high doses (2.5 μg and 5.0 μg) or PBS alone (negative control). The cells were stained with anti-CD4 FITC and anti-CD3 PE antibodies and the CD4^+^ T cells frequency was determined by flow cytometry. The results are expressed in percentage and represent the mean ± SD; the differences were considered significant when p < 0.05 (*) or p < 0.001 (***) compared to PBS control group.
(TIF)

S3 Fig Relative expression of TLR2 in the spleen cells of naïve BALB/c mice after ArtinM administration. Total RNA was extracted from the spleen cells harvested at day 0 and was reverse-transcribed into cDNA. The relative expression of TLR2 was determined by real-time quantitative PCR for mice receiving ArtinM at the specified doses, PBS (negative control), or LPS (positive control). The values were normalized to β-actin expression. Results are expressed as mean ± SD, and the levels of relative expression were compared to the PBS control group. Differences were considered significant when p < 0.05 (*) compared to the PBS control group.
(TIF)
