# Supplementary figures and images for: Systemic effects in naïve mice injected with immunomodulatory lectin ArtinM
Source: PLoS One. 2017 Oct 30;12(10):e0187151. doi: 10.1371/journal.pone.0187151 (PMC5662225; doi:10.1371/journal.pone.0187151)

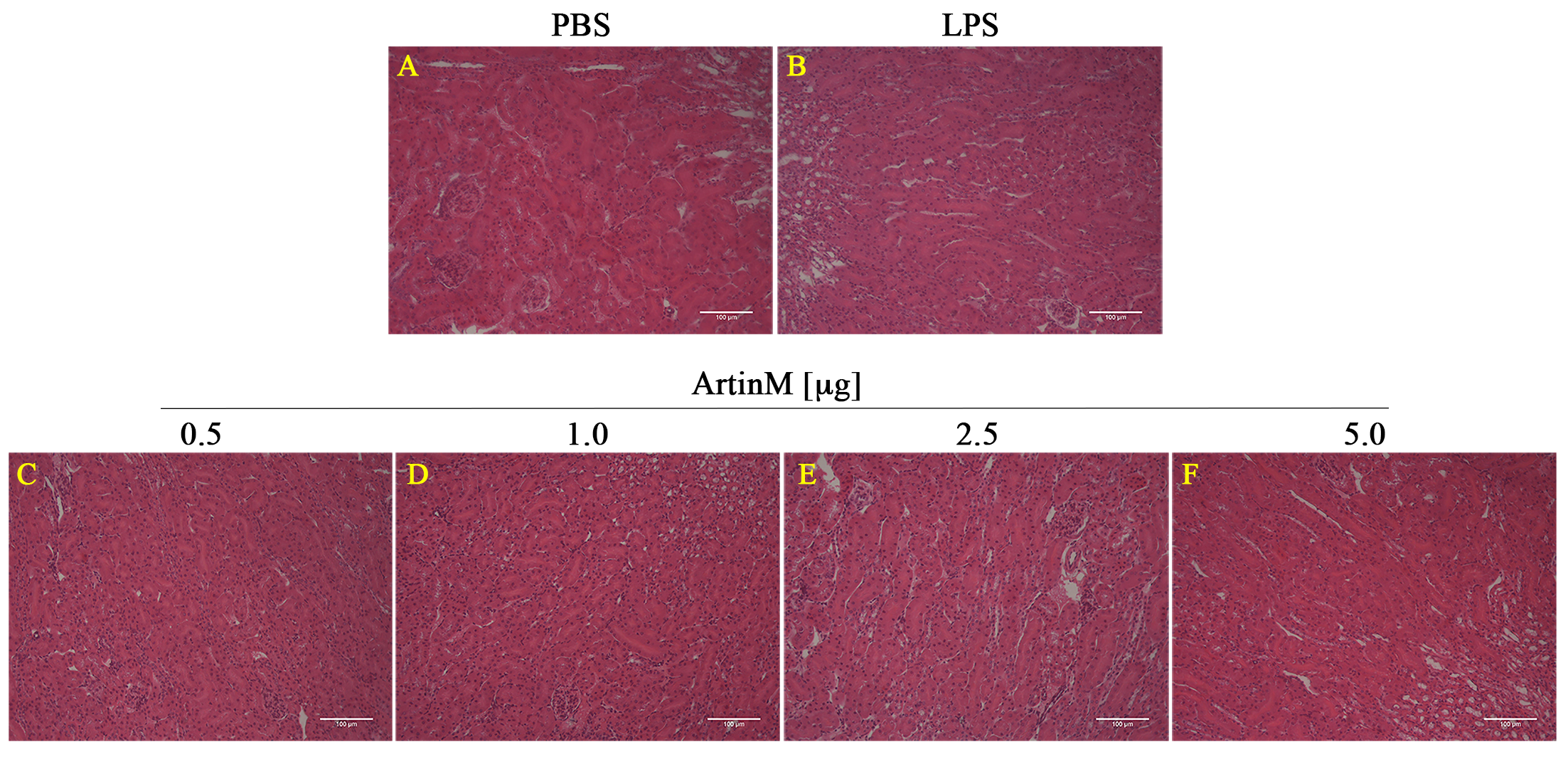

Supplement: S1 Fig — The panels show representative images of kidney sections harvested at day 0 from mice receiving ArtinM at the specified doses, PBS (negative control), or LPS (positive control). The sections were stained with hematoxylin and eosin (H&E), and images were captured using a microscope (Nikon Eclipse 50i) coupled to a digital camera (Evolution MP 5.0). Magnification bars = 100 μm for all sections. (TIF) [file pone.0187151.s001.tif]

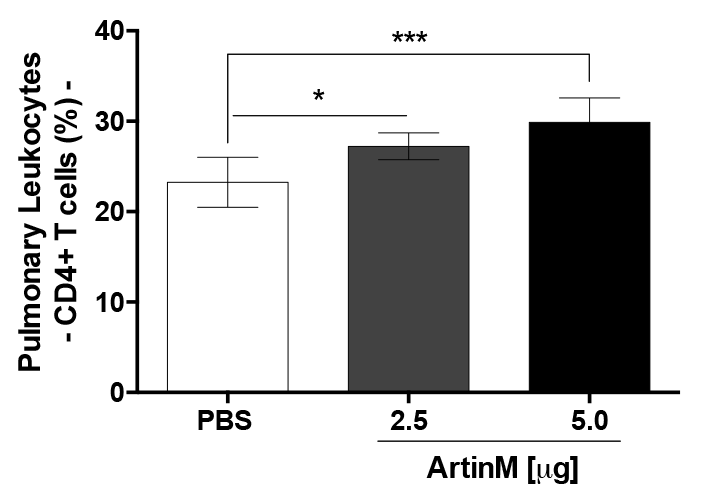

Supplement: S2 Fig — Pulmonary leukocytes harvested from naive mice at day 0 following administration of ArtinM at high doses (2.5 μg and 5.0 μg) or PBS alone (negative control). The cells were stained with anti-CD4 FITC and anti-CD3 PE antibodies and the CD4+ T cells frequency was determined by flow cytometry. The results are expressed in percentage and represent the mean ± SD; the differences were considered significant when p < 0.05 (*) or p < 0.001 (***) compared to PBS control group. (TIF) [file pone.0187151.s002.tif]

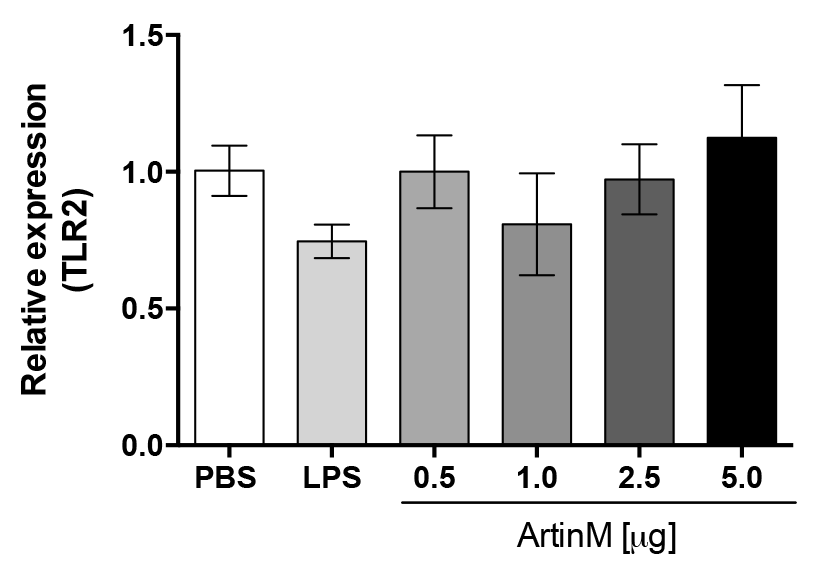

Supplement: S3 Fig — Total RNA was extracted from the spleen cells harvested at day 0 and was reverse-transcribed into cDNA. The relative expression of TLR2 was determined by real-time quantitative PCR for mice receiving ArtinM at the specified doses, PBS (negative control), or LPS (positive control). The values were normalized to β-actin expression. Results are expressed as mean ± SD, and the levels of relative expression were compared to the PBS control group. Differences were considered significant when p < 0.05 (*) compared to the PBS control group. (TIF) [file pone.0187151.s003.tif]
